# Prognostic Impact of Parameters of Metabolic Acidosis in Critically Ill Children with Acute Kidney Injury: A Retrospective Observational Analysis Using the PIC Database

**DOI:** 10.3390/diagnostics10110937

**Published:** 2020-11-11

**Authors:** Hikaru Morooka, Daisuke Kasugai, Akihito Tanaka, Masayuki Ozaki, Atsushi Numaguchi, Shoichi Maruyama

**Affiliations:** 1 Department of Emergency and Critical Care Medicine, Nagoya University Graduate School of Medicine, Nagoya 466-8560, Japan; hikarumorooka@med.nagoya-u.ac.jp (H.M.); nummer@med.nagoya-u.ac.jp (A.N.); 2 Department of Nephrology, Nagoya University Hospital, Nagoya 466-8560, Japan; tanaka17@med.nagoya-u.ac.jp; 3 Department of Emergency and Critical Care Medicine, Komaki City Hospital, Komaki 485-8520, Japan; mozaki@med.nagoya-u.ac.jp; 4 Division of Nephrology, Nagoya University Graduate School of Medicine, Nagoya 466-8560, Japan; marus@med.nagoya-u.ac.jp

**Keywords:** acute kidney injury, pediatric intensive care, sepsis

## Abstract

Acute kidney injury (AKI) is a major complication of sepsis that induces acid-base imbalances. While creatinine levels are the only indicator for assessing the prognosis of AKI, prognostic importance of metabolic acidosis is unknown. We conducted a retrospective observational study by analyzing a large China-based pediatric critical care database from 2010 to 2018. Participants were critically ill children with AKI admitted to intensive care units (ICUs). The study included 1505 children admitted to ICUs with AKI, including 827 males and 678 females. The median age at ICU admission was 22 months (interquartile range 7–65). After a median follow-up of 10.87 days, 4.3% (65 patients) died. After adjusting for confounding factors, hyperlactatemia, low pH, and low bicarbonate levels were independently associated with 28-day mortality (respective odds ratio: 3.06, 2.77, 2.09; *p* values: <0.01, <0.01, <0.01). The infection had no interaction with the three parameters. The AKI stage negatively interacted with bicarbonate and pH but not lactate. The current study shows that among children with AKI, hyperlactatemia, low pH, and hypobicarbonatemia are associated with 28-day mortality.

## 1. Introduction

Acute kidney injury (AKI) is a common problem in both hospitalized adults and children. AKI is well known for its association with increased mortality and adverse outcomes [1,2,3,4]. Recently, new pediatric reference criteria change values optimized for AKI in children (pROCK) were published [5]. Although the pROCK criteria have better precision in identifying children at risk of death, the diagnostic methods for pediatric AKI, not only using pROCK, but also pediatric Risk, Injury, Failure, Loss, End Stage Renal Disease (pRIFLE) and the Kidney Disease Improving Global Outcomes (KDIGO), depend on assessing creatinine changes within 1 week. Therefore, identifying children with high risk factors other than creatinine is necessary for prediction in the earlier phase of the treatment [6,7,8,9].

Sepsis is a major cause of AKI and may deteriorate the prognosis of patients with AKI [10,11]. In patients with sepsis, lactate levels are one of the prognostic markers. Lactate levels have been reflective of cellular dysfunction in sepsis, ischemia in shock or tissue hypoperfusion of any etiology, and hyperlactatemia is now one of the definitions of sepsis in Sepsis-3 [12]. Elevated lactate levels are a sign of hemodynamic insufficiency and are associated with increased mortality in patients with sepsis [13]. AKI is one of the major complications of sepsis and increases lactate levels. Thus, metabolic acidosis is a very complicated pathology in patients with AKI, especially septic AKI.

Metabolic acidosis is fairly easy to evaluate by measuring acid-base balance and has been used as a marker for lactic acidosis, ketoacidosis, rapid volume expansion with saline, renal failure, and others. Metabolic acidosis can be present in many patients with renal injury [14]. Hyperlactatemia is said to be one of the major triggers of metabolic acidosis and in children, the blood lactate level at admission is associated with in-hospital mortality [15]. It is often assumed that hyperlactatemia and metabolic acidosis are associated with worse mortality in both adults and children [16]. However, the parameters of metabolic acidosis are lactate, bicarbonate, and pH. Moreover, which metabolic acidosis-related parameters in critically ill children with AKI are the most predictive of disease outcome is unknown.

In the present study, we hypothesized that among children with AKI who were admitted to intensive care units (ICUs), patients with metabolic acidosis had worse mortality than patients without acidosis. Therefore, we investigated the relationship between prognosis and several acidosis parameters, such as lactate, bicarbonate, and pH. Furthermore, we analyzed how AKI etiology and severity had prognostic interaction with metabolic acidosis parameters regarding AKI outcomes.

## 2. Materials and Methods

### 2.1. Source of Data

The pediatric-specific intensive care database (PIC), a large China-based pediatric critical care database, was analyzed [17]. The PIC is an integrated, de-identified, comprehensive clinical dataset containing routine hospital care records from the Children’s Hospital, Zhjiang University School of Medicine, from 2010 to 2018. The primary cohort contained 12,881 patients, with 13,941 ICU admissions.

### 2.2. Participants

**Figure 1:**
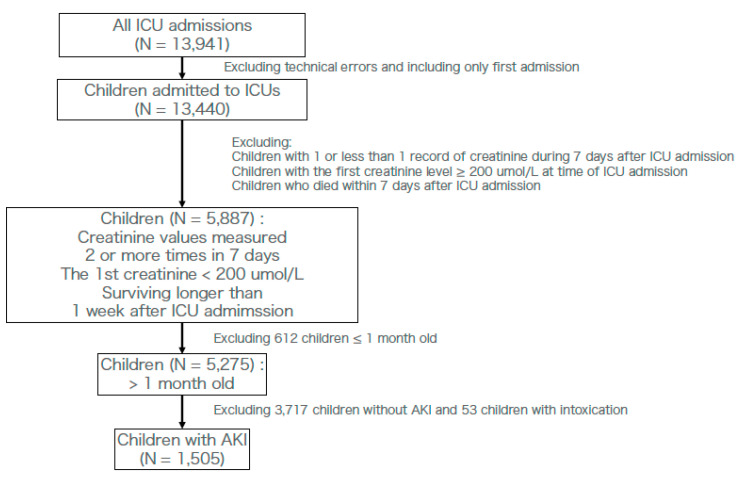
Flow chart of ICU admissions for this observational study. AKI; acute kidney injury. ICU; intensive care unit.

Participants were diagnosed with AKI based on the pROCK criteria during the 7 days after ICU admission. Patient eligibility was considered when creatinine changes met the pROCK criteria within 7 days after ICU admission. Children who were admitted several times were only counted for the first ICU admission, meaning we excluded those ICU admissions that occurred after their 1st ICU admission. Because the original pROCK criteria excluded those whose first creatinine level was >200 µmol/L and those under 1 month of age, we did not include those patients [5]. In order to assess creatinine levels, we excluded the technical errors among them. Patients who died within 7 days in the hospital were excluded because the pROCK criteria required creatinine changes within 7 days (Figure 1) [5]. Furthermore, we excluded children with intoxication by the international classification of diseases 10 (ICD-10) code, which was registered in the original database [18]. We defined the longest follow-up as 28 days.

### 2.3. Diagnosis of AKI

We used the pROCK criterion [5], which defines AKI as an increase in creatinine levels of ≥20 μmol/L and ≥ 30% within 7 days, to diagnose AKI. The pROCK criterion classifies AKI stages 2 and 3 as increases in creatinine levels of ≥40 μmol/L and ≥60% and≥ 80 μmol/L and ≥120%, respectively.

### 2.4. Defining a Cohort with Infection at the Time of ICU Admission

We used the same definition as Sepsis-3 for infection, depending on the use of antibiotics and positive tissue culture [12]. Children fitting the definition, when the various tissue cultures became positive within 24 h before and after ICU admission, were defined as having an infection at the time of ICU admission.

### 2.5. Laboratory Data

Routinely collected clinical and laboratory variables obtained within the first 24 h of ICU admission were used. For the diagnosis of the clinical variables, we used the ICD-10 code, which was registered in the original database [18]. The ICD-10 code was classified according to the ICD-10 code chapters. For variables with multiple measurements, the worst values within the first 24 h of ICU admission were assessed.

### 2.6. Cutoff Values

For this study, we chose three variables: minimum pH, minimum bicarbonate, and maximum lactate levels during the first 24-h ICU stay. We used a spline regression model to evaluate each cutoff value above which the odds ratios were higher than 0.

### 2.7. Outcome

The 28-day mortality was used as the primary outcome. The occurrence of death was based on the original data.

### 2.8. Statistics

Clinical characteristics between the higher lactate and lower lactate groups were compared using a Student’s *t*-test. A Chi-square or Fisher’s exact test was employed to compare the differences in categorical data. Survival was presented graphically using the Kaplan–Meier method. We used multivariable logistic regression analysis to evaluate whether metabolic biomarkers predict mortality. Covariates included in this model were infectious etiology, sex, age, liver function tests (albumin, aspartate transaminase, alanine transaminase, and prothrombin time.), white blood cell, platelets, partial pressure of oxygen. Furthermore, we evaluated the interactions using logistic regression. Because missing data may create bias, variables with >20% missing values were excluded from further analysis. Other variables with a lesser degree of missing values were analyzed using the multiple imputation method [19]. As a sensitivity analysis, we conducted the same analysis on higher bicarbonate levels which can reflect metabolic alkalosis.

*p*-values < 0.05 were considered statistically significant. We used the mice package, the mgcv package, and R software (version 4.0.0, R Foundation for Statistical Computing, Vienna, Austria, URL http://www.R-project.org/) for all statistical analyses [20,21].

## 3. Results

### 3.1. Baseline Characteristics

Of the 13,941 ICU admissions, 5275 children (37.8%) had at least two results of creatinine changes during the first 7 days after ICU admission, initial creatinine levels <200 µmol/L, and were older than 1 month. Among these children, 1505 were diagnosed with AKI based on the pROCK criterion (Figure 1). Patients’ baseline characteristics are shown in Table 1. Missing ratio (%) of laboratory data before multiple imputation is shown in Supplementary Table S1. The population included 1505 children, of whom 827 were male and 678 were female. The median age at ICU admission was 22 months. Among these 1505 children, 413 were diagnosed with infection according to our method. Most of these children had stage 1 AKI (78.8%). Median lactate levels, bicarbonate levels, and mean pH were 2.60 mmol/L, 19.70 mmol/L, and 7.33, respectively. Among these children, the median length of stay in ICUs was 4.84 days, the median length of stay (follow-up) in the hospital was 10.87 days, and the number of children that died within 28 days of admission was 65 (4.3%).

### 3.2. Cut-Off Values

**Figure 2:**
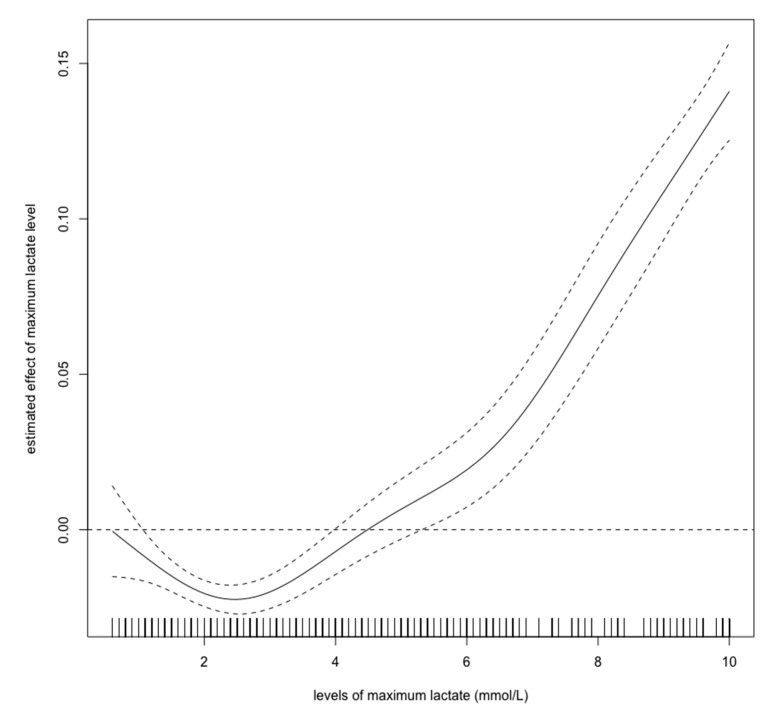
Nonlinear relationship between the lactate level (mmol/L) and 28-day mortality in critically ill children with acute kidney injury. The curve: estimated spline function in log odds ratio on the effect of the lactate level. The dotted lines: 95% confidence interval.

**Figure 3:**
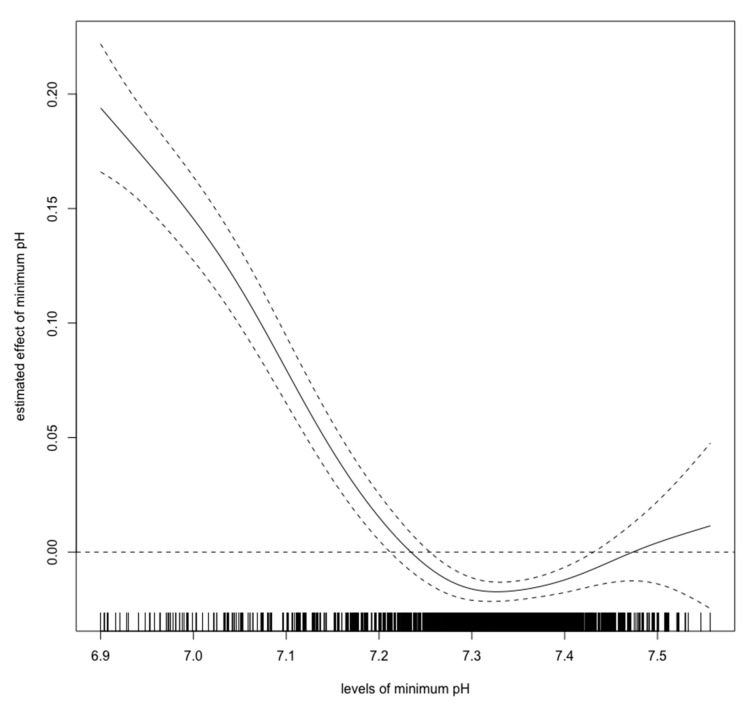
Nonlinear relationship between the pH and 28-day mortality in critically ill children with acute kidney injury. The curve: estimated spline function in log odds ratio on the effect of the pH. The dotted lines: 95% confidence interval.

**Figure 4:**
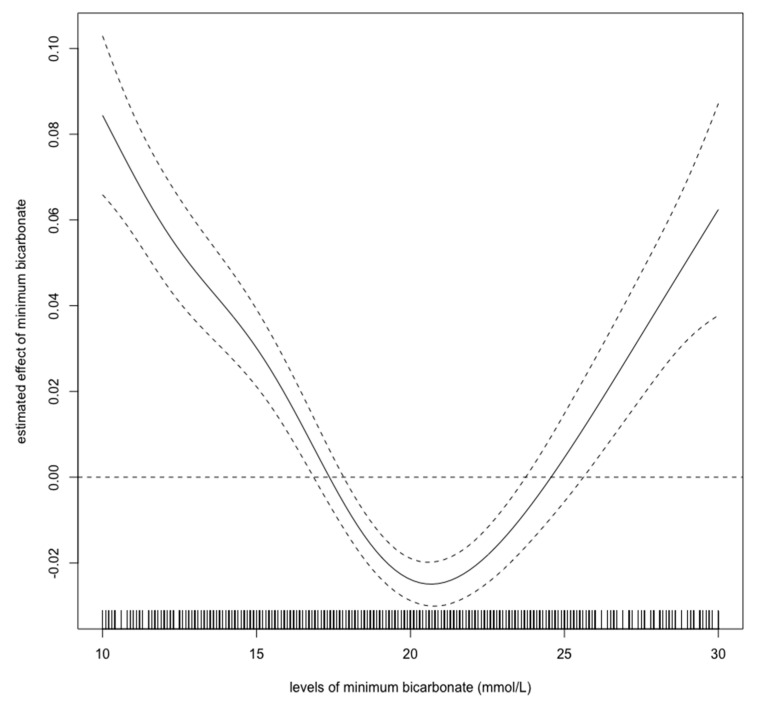
Nonlinear relationship between the bicarbonate level (mmol/L) and 28-day mortality in critically ill children with acute kidney injury. The curve: estimated spline function in log odds ratio on the effect of the bicarbonate level. The dotted lines: 95% confidence interval.

Figure 2, Figure 3 and Figure 4 show estimated non-linear effect of each biomarkers on 28-day mortality. Based on figures, we decided to set cutoff values for each biomarker: lactate, 4.40 mmol/L; pH, 7.25; bicarbonate, 17.5 mmol/L.

### 3.3. Mortality

**Figure 5:**
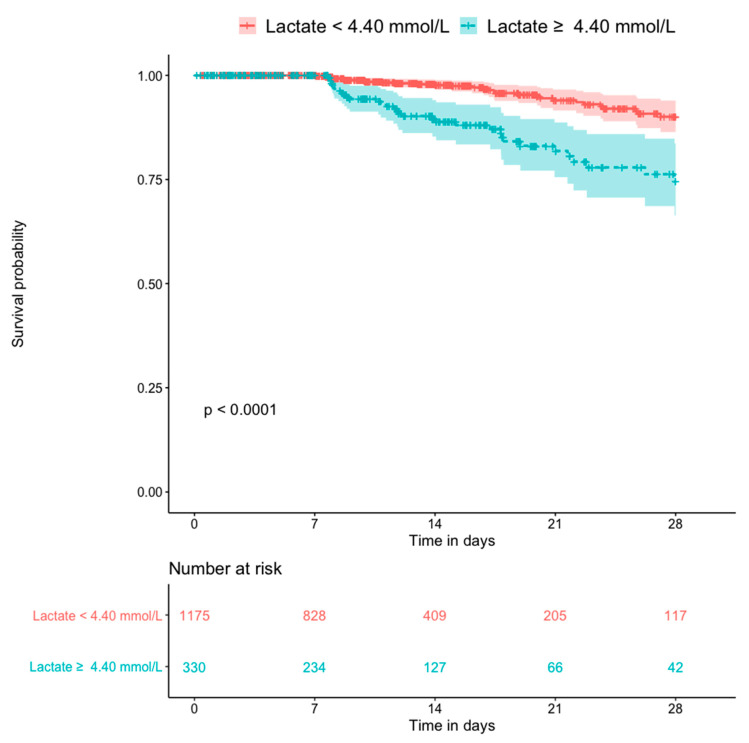
Kaplan–Meier plot for 28-day death compared by the lactate level (n = 1505).

**Figure 6:**
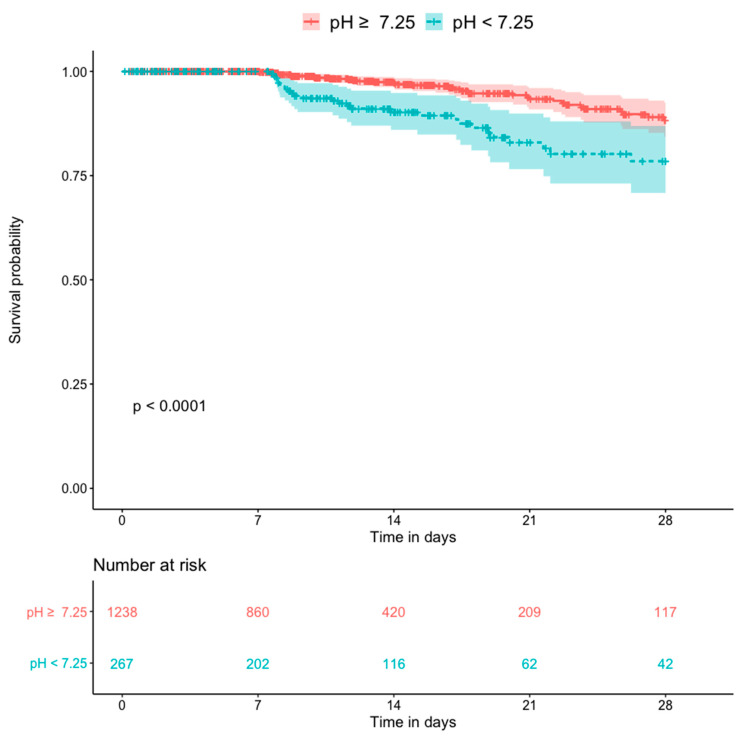
Kaplan–Meier plot for 28-day death compared by the pH (n = 1505).

**Figure 7:**
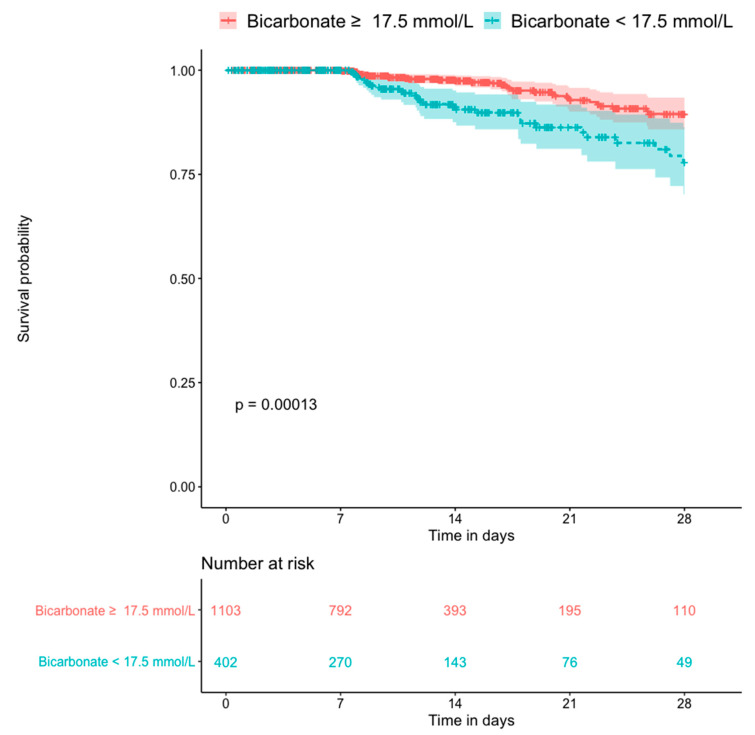
Kaplan–Meier plot for 28-day death compared by the bicarbonate level (n = 1505).

Figure 5 shows the Kaplan–Meier plots for all-cause mortality in patients with and without hyperlactatemia. The former group had a significantly higher mortality rate than the latter group (*p* < 0.0001). Figure 6 shows the Kaplan–Meier plots for all-cause mortality in patients with low pH and high pH. Patients with lower blood pH (<7.25) had a significantly higher mortality rate than those with a higher blood pH (*p* < 0.0001). Figure 7 shows the Kaplan–Meier plots for all-cause mortality in patients with higher and lower bicarbonate levels. Patients with lower bicarbonate levels had a significantly higher mortality rate than those with higher levels (*p* = 0.00013). When bicarbonate levels were divided into three parts (higher, middle, and lower), the higher levels also had significantly higher mortality rate than middle levels (Supplementary Figure S1).

### 3.4. Logistic Regression

The univariate analysis results (Table 2) showed that each laboratory marker (lactate, pH, and bicarbonate) was significantly associated with 28-day mortality (odds ratio [OR]: 3.97, 3.55, 2.63; *p* < 0.01, < 0.01, < 0.01). Table 2 also shows that multivariate analysis did not change this trend (OR: 3.06, 2.77, 2.09; *p* < 0.01, < 0.01, < 0.01). Table 3 shows the interaction effects in the logistic regression model. The interaction effects between infection and each laboratory marker did not show any significant difference (*p*: 0.44, 0.27, 0.19). Table 3 also shows the interaction effects between the AKI stage and each laboratory marker. Maximum lactate (OR: 1.07, *p* = 0.83) was not significantly associated with the AKI stage, while both the pH and bicarbonate level were significantly associated with the AKI stage, meaning that AKI stage can weaken the effect of both laboratory markers (respectively, ORs: 0.52, 0.51; *p* values: 0.04, 0.02). Supplementary Tables S2 and S3 show that higher bicarbonate levels were also significantly associated with mortality but not with infection and pROCK stage.

## 4. Discussion

Our study showed that critically ill children with AKI had a worse prognosis when their lactate levels were higher, pH were lower, and bicarbonate levels were lower. The presence of infection in children with AKI did not change the significance of these laboratory markers. As the AKI stage increases, the importance of these laboratory markers, such as bicarbonate and pH (but not lactate) can be weakened.

Previous studies have shown that hyperlactatemia in critically ill children is associated with mortality, with a cutoff value of approximately 5.55 mmol/L [15]. Our results showed that hyperlactatemia is also related to a worse prognosis. However, the cutoff value for lactate was lower than in the previous report. As Figure 2 shows, the cutoff value for lactate should be 4–5 mmol/L, which is consistent with the previous cutoff value. An elevated initial serum lactate level was reported to be predictive of AKI, and further, mortality in emergency departments in septic patients [22]. In contrast, there are few reports regarding the relationship between pediatric AKI and metabolic acidosis. Our spline models showed that the cutoff values for bicarbonate and pH were 17.5 mmol/L, and 7.25, respectively. The bicarbonate assessment provides the same diagnostic performance as the anion gap assessment and can estimate the presence of metabolic alkalosis and hyperchloremic metabolic acidosis. Measuring bicarbonate is said to be clinically useful [23]. According to the Henderson–Hasselbalch equation, 20.0 mmol/L bicarbonate and a pH of 7.2 are the criteria for metabolic acidosis [24]. Thus, our cutoff values have a high degree of validity. A previous study showed that in children > 1 month of age, mechanical ventilation, hypoxia, AKI, septic shock, malignancy, chronic heart disease, and dialysis are independent risk factors for mortality; however, parameters associated with acidosis were not described in detail [12]. Our results show that in children >1 month of age, metabolic acidosis during the first 24 h in ICU should be a warning for short-term death. Our results are novel, because we identified certain cutoff values that are associated with 28-day mortality in children with AKI.

In this study, among children with AKI, those with hyperlactatemia or metabolic acidosis had a worse prognosis than those without them. The infection did not interact with the prognosis in the three markers associated with acidosis. Whether sepsis changes the prognosis of patients with AKI is controversial [10,11]. Our results did not show any significant difference in mortality, depending on sepsis.

Our results indicate that the AKI stage interacts with the markers, such as bicarbonate and pH, but not with lactate. AKI causes acidosis and increases other types of acids at the same time [25]. Therefore, factors such as bicarbonate and pH could be influenced by these unknown acids, and lactate could not be influenced by these acids created by AKI. The influence of AKI on lactate is less than on the other two markers. The AKI stage is indeed important in assessing the prognosis of children with AKI because the progression of AKI stage showed high mortality. Among the three laboratory markers, the maximum lactate level had the highest odds ratio in both the univariate and multivariate logistic regression models. Lactate levels can be the simplest laboratory marker among the three, because lactate has the highest odds ratio, and AKI affects lactate the least. However, the other two markers were good predictors of 28-day mortality. These two markers, bicarbonate and pH can be influenced by renal failure. As previous studies have also demonstrated relationships between hyperlactatemia and mortality, our results indicate that our analyses were valid [11,15].

The strengths of our study include the large number of participants and accuracy of AKI diagnosis. Furthermore, our results suggest that in addition to creatinine, biomarkers such as lactate, bicarbonate, and pH can be effective in detecting all-cause mortality in children with critical AKI at the time of admission. Our findings can be generalized to the Asian population with any etiology of AKI.

There are several limitations to this study. First, the original database does not include information about the use of a respirator and dialysis therapy. Second, the original database is based on retrospective real-world data, meaning that unrecorded factors could interact with our analyses. Third, because we excluded cases with death within 1 week due to the pROCK definition, we might have missed critical cases. The estimated effect of sepsis could also be underestimated.

## 5. Conclusions

The current study shows that hyperlactatemia, bicarbonate, and pH are associated with 28-day mortality among children with AKI. The presence of infection does not have an influence on these markers. The prognostic impact of bicarbonate and pH is interacted with the AKI stage.

## 6. Ethics Approval and Consent to Participate

Data used in this study were de-identified in accordance with Health Insurance Portability and Accountability Act (HIPAA) standards [26]. Therefore, the ethical approval statement by the institutional review board and informed consent were waived for this study.

## Figures and Tables

**Table 1:** Baseline characteristics of children admitted to intensive care unit (N = 1505).

Male, n (%)	827 (55.0)
Age, month, median (IQR)	22 (7–65)
AKI severity ^a^, n (%)	
Stage 1	1186 (78.8)
Stage 2	172 (911.4)
Stage 3	147 (9.8)
Primary diagnosis on ICU admission ^b^, n (%)	
Hematological	60 (4.0)
Circulation	146 (9.7)
Congenital	450 (29.9)
Digestive	77 (5.1)
Endocrinology	39 (2.6)
Genitourinary	27 (1.8)
Infectious	10 (0.7)
Musculoskeletal	13 (0.9)
Neoplasm	15 (1.0)
Respiratory	173 (11.5)
Others	495 (32.9)
Infectious etiology of AKI, n (%)	413 (27.4)
Laboratory data ^c^	
Albumin, g/L, median (IQR)	38.10 (33.40–42.00)
Alanine transaminase, U/L, median (IQR)	33.00 (23.00–58.00)
Aspartate transaminase, U/L, median (IQR)	78.00 (41.00–150.00)
Total bilirubin, µmol/L, median (IQR)	12.20 (7.20–24.90)
Potassium, mmol/L, median (IQR)	3.30 (2.90–3.60)
Chloride, mmol/L, median (IQR)	112.00 (108.00–116.00)
Sodium, mmol/L, median (IQR)	136.00 (133.00–139.00)
Phosphate, mmol/L, median (IQR)	1.61 (1.28–2.12)
Base Excess, mmol/L, median (IQR)	−4.80 (-7.60–-2.50)
Creatinine, μmol/L, median (IQR)	51.00 (42.00–64.00)
Urea, mmol/L, median (IQR)	4.34 (3.14–6.13)
White blood cell, ×10^9^/L, median (IQR)	12.66 (8.69–17.57)
Hemoglobin, g/L, median (IQR)	101.00 (88.00–113.00)
Platelets, ×10^9^/L, median (IQR)	191.00 (109.00–282.00)
Prothrombin time, second, median (IQR)	14.30 (12.60–17.10)
Partial pressure of oxygen_,_ mmHg, median (IQR)	83.50 (47.80–132.00)
Lactate, mmol/L, median (IQR)	2.60 (1.70–4.00)
pH, median (IQR)	7.33 (7.28–7.38)
Bicarbonate, mmol/L, median (IQR)	19.70 (17.20–22.00)
Length of ICU stay, day, median (IQR)	4.84 (1.87–11.15)
Length of hospital stay, day, median (IQR)	10.87 (6.75–17.34)
28-day mortality, n (%)	65 (4.3)

^a^: acute kidney injury defined by pROCK criteria. ^b^: according to the International Classification of Diseases 10. ^c^: the worst value in the first 24-h after ICU admission. AKI, acute kidney injury; ICU, intensive care unit; IQR, interquartile range; SD, standard deviation; pROCK, pediatric reference change value optimized for acute kidney injury in children.

**Table 2:** Predictors of 28-day mortality in children with acute kidney injury by uni- and multivariate logistic regression.

	Crude OR (95% CI)	Adjusted OR (95% CI)	*p* Value
Lactate ≧ 4.40 mmol/L	3.97 (2.40–6.56)	3.06 (1.78–5.26)	<0.01
pH < 7.25	3.55 (2.13–5.93)	2.77 (1.60–4.81)	<0.01
Bicarbonate < 17.5 mmol/L	2.63 (1.59–4.34)	2.09 (1.23–3.54)	<0.01

OR, Odds ratio; CI, 95% confidence interval. Other factors included infection, sex, age (month old), albumin, aspartate transaminase, alanine transaminase, white blood cell, platelets, partial pressure of oxygen, and prothrombin time.

**Table 3:** Interactions of 28-day mortality in children with acute kidney injury by logistic regression.

	OR (95% CI)	*p* Value
Interactions between parameters and infection		
Lactate × infection	0.66 (0.23–1.88)	0.44
pH × infection	0.54 (0.19–1.59)	0.27
Bicarbonate × infection	0.50 (0.18–1.42)	0.19
Interactions between parameters and AKI severity		
Lactate × pROCK	1.07 (0.59–1.95)	0.83
pH × pROCK	0.52 (0.28–0.96)	0.04
Bicarbonate × pROCK	0.51 (0.29–0.91)	0.02

OR, Odds ratio; CI, 95% confidence interval; AKI, Acute kidney injury; pROCK, pediatric reference change value optimized for acute kidney injury in children.

## Supplementary Materials

The following are available online at https://www.mdpi.com/2075-4418/10/11/937/s1, Supplementary table S1: Missing ratio (%) of laboratory data before multiple implementation. Supplementary Figure S1: Kaplan-Meier plot for 28-day death compared by the bicarbonate level (n = 1505). Supplementary Table S2: Predictors of 28-day mortality in children with acute kidney injury by uni- and multivariate logistic regression. Supplementary Table S3: Interactions of 28-day mortality in children with acute kidney injury by logistic regression.
