# Supplementary material for: Prognostic Impact of Parameters of Metabolic Acidosis in Critically Ill Children with Acute Kidney Injury: A Retrospective Observational Analysis Using the PIC Database
Source: Diagnostics (Basel). 2020 Nov 11;10(11):937. doi: 10.3390/diagnostics10110937 (PMC7696045; doi:10.3390/diagnostics10110937)
Supplement: Supplementary file 1 [file diagnostics-10-00937-s001.pdf]

Supplementary Materials:

Supplementary Table S1: Missing ratio (%) of laboratory data before multiple implementation

ALT	Alb	AST	Base Excess
5.5	16.5	16.6	1.7
WBC	TBil	Bicarbonate	Cl
2.2	16.4	1.0	1.0
Creatinine	Hemoglobin	Lactate	pH
5.5	2.2	2.7	1.0
Phosphate	Plt	pO_2	Potassium
17.1	2.2	1.0	1.1
PT	Sodium	Urea	
9.2	1.0	5.3	

ALT, alanine transaminase; Alb, albumin; AST, aspartate transaminase; TBil, total bilirubin; Cl, chloride; Plt, platelets; pO_2, partial pressure of oxygen; PT, prothrombin time; WBC, white blood cell; Cre, creatinine; LOS, length of stay in intensive care unit

Supplementary Figure S1: Kaplan-Meier plot for 28-day death compared by the bicarbonate level (n = 1,505)

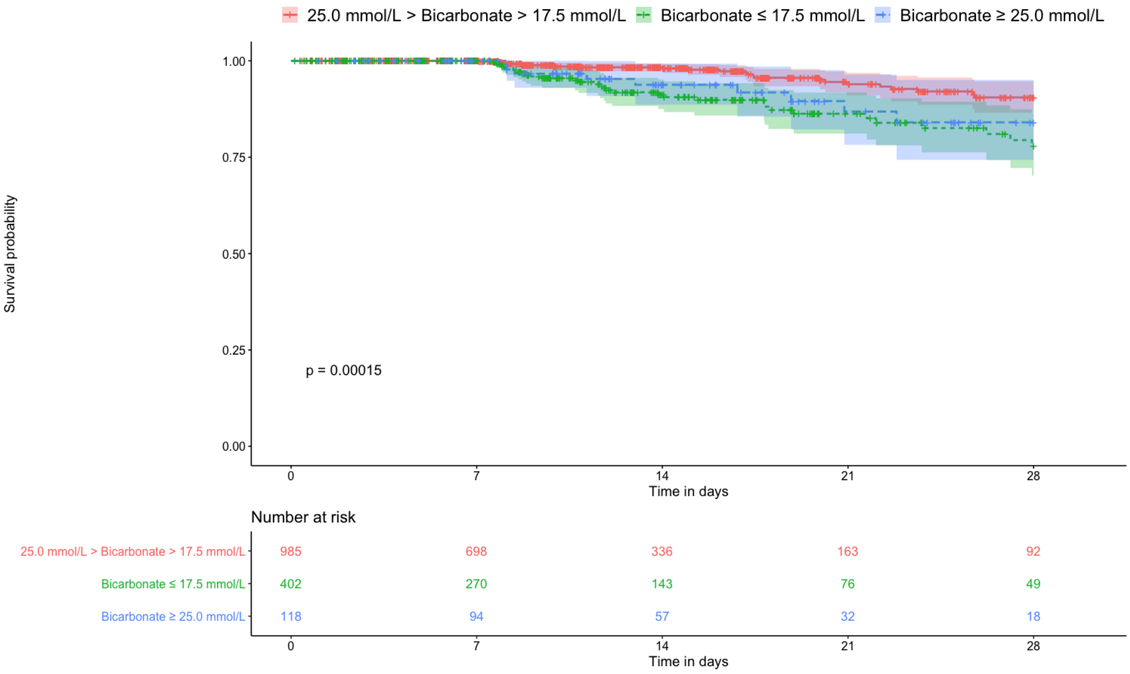

Supplementary Table S2: Predictors of 28-day mortality in children with acute kidney injury by uni- and multivariate logistic regression

	**Crude OR (95% CI)**	**p value**	**Adjusted OR (95% CI)**	**p value**

Bicarbonate > 17.5 and < 25.0 mmol/L	Reference	Reference	Reference	Reference
Bicarbonate ≦ 17.5 mmol/L	3.21 (1.87 - 5.51)	< 0.01	2.51 (1.43 - 4.42)	< 0.01
Bicarbonate ≧ 25.0 mmol/L	3.17 (1.44 – 6.97)	< 0.01	2.82 (1.26 - 6.30)	0.01

OR, Odds ratio; CI, 95% confidence interval.

Other factors included infection, sex, age (month old), albumin, aspartate transaminase, alanine transaminase, white blood cell, platelets, partial pressure of oxygen, and prothrombin time.

Supplementary Table S3: Interactions of 28-day mortality in children with acute kidney injury by logistic regression

	**OR (95% CI)**	**P value**
Interactions between biomarkers and AKI severity		
Bicarbonate > 17.5 and < 25.0 mmol/L × infection	Reference	Reference
Bicarbonate ≦ 17.5 mmol/L × infection	0.44 (0.14 - 1.35)	0.15
Bicarbonate ≧ 25.0 mmol/L × infection	0.47 (0.09 - 2.49)	0.38
Interactions between biomarkers and AKI etiology		
Bicarbonate > 17.5 and < 25.0 mmol/L × pROCK	Reference	Reference
Bicarbonate ≦ 17.5 mmol/L × pROCK	0.56 (0.30 - 1.04)	0.07
Bicarbonate ≧ 25.0 mmol/L × pROCK	2.18 (0.73 - 6.50)	0.16

OR, Odds ratio; CI, 95% confidence interval;AKI, Acute kidney injury; pROCK, pediatric reference change value optimized for acute kidney injury in children
